# Fusion Attention Mechanism for Foreground Detection Based on Multiscale U-Net Architecture

**DOI:** 10.1155/2022/7432615

**Published:** 2022-09-19

**Authors:** Peng Liu, Junying Feng, Jianli Sang, Yong Kwan Kim

**Affiliations:** ^1^School of Intelligent Manufacturing, Weifang University of Science and Technology, Weifang 261000, China; ^2^Department of Information and Communication Engineering, Hoseo University, Asan 31499, Republic of Korea

## Abstract

Foreground detection is a classic video processing task, widely used in video surveillance and other fields, and is the basic step of many computer vision tasks. The scene in the real world is complex and changeable, and it is difficult for traditional unsupervised methods to accurately extract foreground targets. Based on deep learning theory, this paper proposes a foreground detection method based on the multiscale U-Net architecture with a fusion attention mechanism. The attention mechanism is introduced into the U-Net multiscale architecture through skip connections, causing the network model to pay more attention to the foreground objects, suppressing irrelevant background regions, and improving the learning ability of the model. We conducted experiments and evaluations on the CDnet-2014 dataset. The proposed model inputs a single RGB image and only utilizes spatial information, with an overall F-measure of 0.9785. The input of multiple images is fused, and the overall F-measure can reach 0.9830 by using spatiotemporal information. Especially in the Low Framerate category, the F-measure exceeds the current state-of-the-art methods. The experimental results demonstrate the effectiveness and superiority of our proposed method.

## 1. Introduction

Intelligent video surveillance plays an important role in the fields of transportation, security, and industrial production. It is very necessary for the foreground detection of targets such as people, animals, and vehicles. As the underlying task of intelligent video surveillance, foreground detection is an important basis for subsequent high-level tasks such as target tracking, target recognition, and behavior analysis. The quality of its detection effect directly affects the performance of subsequent tasks. Foreground detection, also known as foreground segmentation, is one of the research hotspots in the field of computer vision. Its application is not limited to intelligent video surveillance but is widely used in human-computer interaction [1], video coding [2], automatic driving [3], and other fields. In practical application scenarios, there are often many interference factors such as dynamic background, camera jitter, illumination changes, and shadows, so it is very important to study more robust and efficient foreground detection methods.

**Figure 1:**
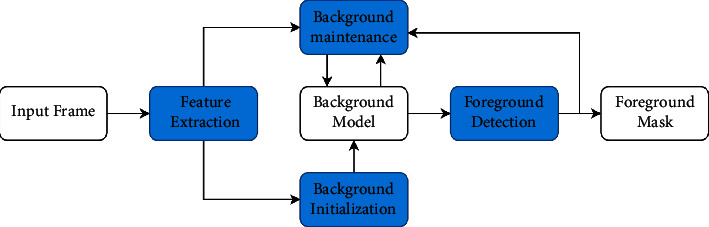
Steps of background modeling method.

Foreground detection methods are generally divided into three categories: optical flow method, inter-frame difference method, and background modeling method. The optical flow method can adapt to changes in dynamic scenes, but it is difficult to use in practice due to the high complexity of the algorithm. The inter-frame difference method has low algorithm complexity and is not very sensitive to the illumination changes in the scene, but there will be a lot of holes in the detected foreground objects, which will affect the detection effect. The background modeling method is currently the mainstream method and is the most widely used. It usually has the following four steps: feature extraction, background model initialization, background model maintenance, and foreground detection (see Figure 1). Generally, a background model is established by designing a feature algorithm, the current input image and the background model are compared, the image is divided into foreground pixels and background pixels according to the threshold, and the result is represented by a binary image. The performance of its foreground detection largely depends on the accuracy of the background model. Traditional background modeling methods are generally based on hand-crafted features and usually rely on strong prior conditions. They only perform well in specific types of scenes, are usually difficult to adapt to various interference challenges, and perform poorly in complex scenes.

In recent years, with the rapid development of deep learning technology, convolutional neural networks (CNN) [4] have been proven to be able to effectively learn deep abstract features and have achieved great success in computer vision fields such as image classification, image segmentation, and object detection. The purpose of foreground detection is to separate the foreground objects from the background, which is a typical pixel-level binary classification problem. It is also an image segmentation task. Compared with traditional background modeling methods, CNN can learn from training data to obtain powerful feature extraction capabilities, and the extracted features are often better than hand-crafted features, which can significantly improve the effect of foreground detection.

In 2015, Long et al. [5] proposed a fully convolutional network (FCN). By replacing the fully connected layers of the VGG [6] network with convolutional layers, dense prediction at the pixel level of the image is achieved. For some application scenarios with strict segmentation accuracy requirements, such as medical image segmentation, Ronneberger et al. [7] proposed a multiscale fully convolutional neural network, U-Net, that can fuse shallow and deep features. The feature maps of different scales in the encoding network are passed to the decoding network of the corresponding size through skip connections and are concatenated with the feature map channels of the decoding network to achieve multiscale feature fusion. Oktay et al. [8] added an attention mechanism to the U-Net network, which better realized the attention to salient regions and suppressed irrelevant background regions, and obtained satisfactory results.

Inspired by the above research work, this paper proposes a model with a fusion attention mechanism based on the multiscale U-Net architecture for foreground detection. We name the model AMU-Net, which is an end-to-end encoder-decoder structure. The encoder adopts a pretrained VGG-16 [6] network for downsampling to extract feature information, and the decoder learns the mapping from feature space to image space by upsampling with transposed convolutions. The attention mechanism is added to the skip connections between the encoder and the decoder so that the network learns more features related to the foreground target and suppresses the learning of background features that are irrelevant to the task. We test and evaluate the model on the CDnet-2014 dataset [9], and the results show that the proposed method outperforms most existing methods. The main contributions of our work are as follows:

1. We propose a network model based on a multiscale feature fusion attention mechanism for foreground detection, which requires only a small amount of training data and only uses image spatial information to achieve accurate foreground segmentation.
2. We conducted two types of multi-input experiments, which not only utilized spatial information but also added time-varying information to further improve the detection performance of the model. Especially in the very challenging Low Framerate category, the multi-input methods show excellent results.

The rest of this paper is organized as follows.  Section 2 briefly reviews related work.  Section 3 details the proposed model architecture.  Section 4 presents the experimental results on the CDnet-2014 dataset and compares them with other state-of-the-art methods. Finally, Section 5 concludes the full text.

## 2. Related Works

In the past three decades, many scholars have proposed various background models and algorithms to improve the foreground detection effect. Bouwmans et al. [10–13] review the existing various foreground detection methods and summarize them well. We mainly review the more representative methods, which can be divided into traditional unsupervised methods and deep learning-based supervised methods according to whether the annotation information is used or not.

### 2.1. Traditional Unsupervised Methods

In 1999, Stauffer and Grimson [14] proposed the classical Gaussian mixture model (GMM), which uses multiple Gaussian distributions to estimate changes in background color with time, but it is difficult to model the rapidly changing background, and more false detection is likely to be generated. Elgammal et al. [15] proposed a background model based on Kernel Density Estimation (KDE). By estimating the video sample data with a kernel function, the sample data with the highest probability density was selected as the background. It is a time-consuming and space-complex method because it needs to store a large amount of historical data. Kim et al. [16] proposed a codebook model, which builds a codebook for each pixel, and each codebook contains a set of codewords. First, the background learning is performed on the video sequence to obtain the background model codebook. When detecting the foreground target, the pixels are matched with the codewords in the corresponding codebook. If the pixel value falls within the corresponding codeword, it is classified as a background pixel; otherwise, it is a foreground pixel. Heikkila and Pietikainen [17] first introduced Local Binary Pattern (LBP) into the foreground detection task. The pixels of the image area are marked by threshold processing of the center pixel and its neighbor pixels, and the result is represented in binary form. Liao et al. [18] designed a Scale Invariant Local Ternary Pattern (SILTP) based on LBP features and used improved KDE to model the probability distribution of SILTP. Barnich and Van Droogenbroeck [19] proposed a nonparametric algorithm called ViBe, which establishes a sample set of background pixels for each pixel in the image as a background model. The matching number of pixels is obtained by comparing the background model samples and the new input image, to determine whether a pixel belongs to the foreground pixels or the background pixels. If it belongs to the background pixels, it is updated to the background model with a certain probability. The ViBe algorithm improves the fault tolerance rate of detection, has better stability, and can adapt to slow background motion changes. Hofmann et al. [20] proposed the PBAS algorithm based on ViBe, which improved the robustness of the model by dynamically adjusting the threshold and the update rate of each pixel. St-Charles et al. proposed the SuBSENSE [21] and PAWCS [22] algorithms, which improved the feature expression ability by combining texture and color features, and used a pixel-level feedback strategy to automatically adjust internal parameters. Wang et al. [23] proposed the FTSG algorithm, which combined a split Gaussian model with a flux tensor (optical flow feature) to improve the detection effect. The algorithm uses a Gaussian mixture model as the background, using a single Gaussian as the foreground; by computing the flux tensor [24], it can account for optical flow variations within a local 3D spatiotemporal volume and is used to detect blob motion. Bianco et al. [25] proposed the IUTIS-5 method, which integrates a variety of state-of-the-art algorithms through genetic coding to deal with complex background scenes.

The above unsupervised traditional methods primarily perform foreground detection with hand-crafted features, which are sensitive to changes in the video scene. Therefore, such methods are generally suitable for some specific or simple video scenes, and their performance will be poor when it faces sudden lighting changes, shadows, camouflage, etc. In addition, although these algorithms are unsupervised, to improve the robustness of the algorithm, a large number of parameters need to be adjusted by humans rather than computers. Therefore, traditional unsupervised methods are not sufficient to handle complex real-world scenarios.

### 2.2. Methods Based on Deep Learning

In 2016, Braham and Van Droogenbroeck [26] first introduced a convolutional neural network in foreground detection. The method first extracts the background image through median filtering in 150 initialization frames, then extracts image blocks centered on each pixel from the current input frame and background image, and finally inputs the image block combination into the trained network model to calculate the foreground probability for this pixel. Babaee et al. [27] proposed an improvement on the generation of background images, which enhanced the background images with the output results of unsupervised algorithms SuBSENSE [21] and FTSG [23]. Wang et al. [28] proposed a multiscale cascaded convolutional neural network. The model has two key structures: (1) In the multiscale structure, the model downsamples the current frame with a ratio of 0.75 and 0.5; then the current frame and the downsampled frame are sent to the CNN model; and finally, the output results of different sizes are upsampled, and average pooling is performed to achieve the segmentation of the foreground image. (2) In the cascade structure, to reduce pixel misclassification caused by local information, the foreground probability map output by the first CNN model and the current image are input to the second CNN model again, and a more refined foreground probability map is output. Different from the above three patch-based convolutional neural networks for prediction, a network architecture based on FCN [5] was proposed by Zeng and Zhu [29] for foreground detection. The whole image is used as the input of the network for multiscale feature extraction, and the contrast layer is used to strengthen the learning of the difference between the foreground target and the background region. Lim and Yalim Keles [30] proposed multiscale segmentation architectures named FgSegNet_M and FgSegNet_S, both of which are encoder-decoder structures. FgSegNet_M uses pretrained VGG-16 [6] as the encoder, and the current image is divided into three different scales and input into three sets of encoders, which are decoded using a transposed convolutional neural network to obtain the final segmentation map. FgSegNet_S only keeps a set of encoders and uses a feature pooling module (FPM) with atrous convolution to extract multiscale information. In further research, they propose an improved [31] architecture, FgSegNet_v2, which modifies the FPM and adds skip connections between the encoder and decoder. This algorithm currently ranks first on the CDnet-2014 dataset [9], outperforming all other methods. Tezcan et al. [32] proposed a BSUV-Net model for foreground detection of unseen videos. The method uses a U-Net [7] type fully convolutional neural network with skip connections. The input to the network consists of the current frame and two background frames at different time scales and their semantic information. Although the proposed method has certain generalizations, the performance of the method needs to be improved in challenging situations such as camera jitter and dynamic background. Sakkos et al. [33] first used 3D convolutional neural networks [34] to build a background subtraction model, spatiotemporally encoding video sequences and capturing the features of spatial dimensions and changes in temporal dimensions. Hu et al. [35] and Akilan et al. [36] both use 3D convolutional layers and ConvLSTM layers to extract spatiotemporal information. Zheng and Wang [37] proposed a foreground detection model, BSGAN, based on Bayesian Generative Adversarial Network (Bayesian GAN) [38]. BSGAN uses convolutional neural networks to build generators and discriminators and conduct adversarial training, so that the generator obtains the ability to segment foreground and background. To improve the robustness of the model, Zheng et al. further proposed BSPVGAN [39], which introduced the parallel vision theory on the basis of BSGAN to improve the foreground detection effect of complex scenes.

The features that need to be extracted in many complex scenes in the foreground detection task are extremely challenging, and the hand-crafted features cannot meet the requirements, while the deep learning method can extract the required abstract features from the data and figure out the feature mapping between the input and output data, which can solve complex computer vision problems.

## 3. The Proposed Method

**Figure 2:**
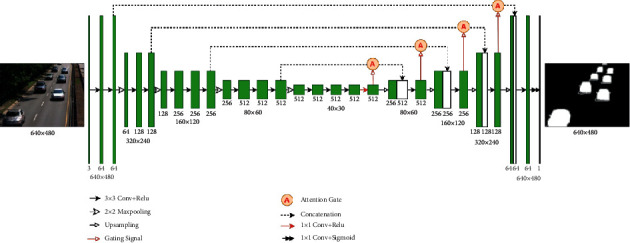
The architecture of the proposed AMU-Net for foreground detection.

The proposed AMU-Net architecture is shown in Figure 2, which is an end-to-end fully convolutional encoder-decoder network. We divide it into three parts: encoder network, decoder network, and attention mechanism module. The encoder network aims to learn more semantic information by gradually reducing the spatial size of the feature maps, the decoder network restores the spatial size of the feature maps through an upsampling operation, and the skip connections fused with the attention mechanism capture the local and global context information on features at different resolutions (scales), resulting in more accurate foreground detection results. The input of the network is a three-channel RGB image, and the output of the network is a foreground probability map of the same size as the input. This method does not need to pre-extract the background image.

### 3.1. Encoder-Decoder Network

As shown in Figure 2, the encoder network in the model is a VGG-16 network. Because we only use a small amount of training data in the experiments, we use the transfer learning method and use VGG-16 pretrained on the ImageNet dataset [40]. Transfer learning is widely used in many fields [41, 42]. By initializing with a pretrained network and then fine-tuning the weights on the new network, this method can achieve faster convergence than training a new network with the random initialization and generally obtain higher accuracy. Through a series of convolution and pooling operations in the encoder network, the feature map is downsampled four times, and the size of the corresponding feature map is shown in Figure 2. It can be seen that the spatial resolution of the lower layer is higher; as the feature layer deepens, its spatial resolution continues to decrease; and the encoder achieves high-level semantic coding by continuously reducing the resolution while increasing the number of feature maps. We made some modifications to the VGG-16 network to make it suitable for the task of this paper. We removed the last pooling layer and all fully connected layers of the VGG-16 network. Although the fully connected layer contains more high-level semantic information, due to the lack of spatial details, it is not suitable for pixel-level foreground detection tasks, and the calculation cost of the fully connected layer is very high. Some recent studies [6, 42] have shown that features extracted from different convolutional layers have different roles. The resolution of the lower layer is relatively high, but only the local details of the image are perceived, while the deeper layers can obtain the global context information of the image, but the resolution is lower. Not only can the use of depth feature information from different layers get more accurate location information, but it also preserves high-level semantic information. Therefore, to fuse the multiscale features of different feature layers, we extract the convolution output before each pooling layer, fuse the attention mechanism through skip connections, and concatenate the corresponding convolutional layers of the decoder network together. That way, the detected foreground target boundary information is more complete, and the final foreground target is more accurate.

The resolution of the AMU-Net input image is uniformly adjusted to 640 × 480, and after feature extraction by the VGG-16 encoder, 512 feature maps with a resolution of 40 × 30 are finally generated. These feature maps are fed into the decoder network through 1 × 1 convolution. The decoder network is divided into four stages, and the feature maps of each stage are upsampled and then combined with the low-level features of the encoder passed through skip connections. This way, multiscale information from different feature layers is obtained. Upsampling is achieved by transposed convolution. To reduce the checkerboard effect [43], the transposed convolution of size 4 × 4 and stride 2 is used to enlarge the resolution of the feature map. The size is gradually enlarged with the upsampling; the concatenated feature maps are successively passed through the 3 × 3 convolution and ReLU activation function; the final network output size is consistent with the input size; and finally, the number of feature maps is reduced to 1 through 1 × 1 convolution. The decoder achieves foreground/background semantic decoding by continuously reducing the number of feature maps while increasing the resolution. The final output layer of the decoder uses the sigmoid activation function to map the features between 0 and 1 to generate a foreground probability map, and the probability map is binarized to obtain the foreground/background segmentation masks.

### 3.2. Attention Mechanism Module

**Figure 3:**
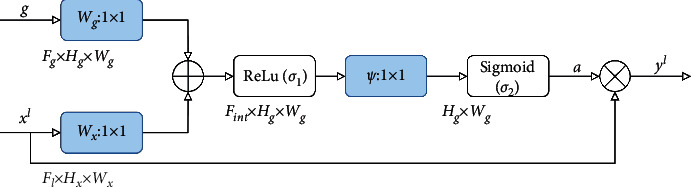
The internal structure of the attention mechanism module.

The attention mechanism [44] in the field of deep learning is essentially similar to the human visual selective attention mechanism, which tends to focus on some specific parts of all the information that has been collected. Considering the advantages of this mechanism in discrimination and focusing, the attention mechanism has been widely used in various fields of artificial intelligence. In the standard U-Net network of encoding-decoding mode, although there is more spatial information in the shallow network, the learned features are not as rich as the deep ones. In addition, similar shallow features should not be extracted repeatedly when using skip connections for feature fusion; otherwise, it will lead to computational burden and model parameter redundancy. Therefore, this paper applies the attention mechanism to skip connections to increase the weight of foreground objects to suppress the interference of background pixels, thereby improving the learning ability of the model. The internal structure of the attention mechanism module is shown in Figure 3.

The module has two inputs: one is the upsampled feature *g*(*F*_*g*_ × *H*_*g*_ × *W*_*g*_), and the other is the encoded feature *x*^*l*^(*F*_*l*_ × *H*_*x*_ × *W*_*x*_) with the same resolution transmitted through skip connections, where the upsampled feature *g* can be regarded as a gating signal that enhances the learning ability of *x*^*l*^. The two inputs are first subjected to a 1 × 1 convolution operation to obtain *W*_*g*_^*T*^*g*_*i*_ and *W*_*x*_^*T*^*x*_*i*_^*l*^. Then, the two results are added, and then ReLU (*σ*_1_(*x*_*i*_^*l*^)=max(0, *x*_*i*_^*l*^)) activation is performed. The fused features will undergo convolution operation again, and through the activation function sigmoid (*σ*_2_), the attention coefficient *a* is obtained; that is,

(1) $$\begin{array}{c} q_{\text{att}}^{l}=\varphi^{T}\left(\sigma_{1}\left(W_{x}^{T}x_{i}^{l}+W_{g}^{T}g_{i}+b_{g}\right)\right)+b_{\varphi },\\ a=\sigma_{2}\left(q_{\text{att}}^{l}\left(x_{i}^{l},g_{i}\right)\right), \end{array}$$

where *b*_*g*_ and  *b*_*φ*_ represent the bias term and *φ* represents the convolution kernel of size 1 × 1. Finally, the encoded features are multiplied by the attention coefficient *α* to output a new feature map *y*^*l*^.

### 3.3. Loss Function

A common problem in foreground detection tasks is that there are far more background pixels in the scene than foreground pixels; this problem is also known as the class imbalance problem. We take foreground pixels as positive samples and background pixels as negative samples.

The class imbalance problem has two consequences:

1. There is extremely unbalanced proportion of positive and negative samples.
2. The number of negative samples greatly exceeds that of positive samples, and many categories of negative samples are relatively easy to identify.
3. The difficulty of sample classification is unbalanced.

For the negative samples that are easier to identify, although their loss values are not high, when the number of samples is very large, the superposition of these loss values will have a very large impact on the final total loss value. The gradient optimization process of training is over-influenced by easily identifiable negative samples, so that it pays too much attention to these loss values and eventually converges to an insufficiently good result.

In order to suppress the loss caused by a large number of easy-to-learn background samples and prevent the network from being misled, this paper adopts a loss function composed of binary cross-entropy loss (2) and Tversky loss [45] (3) to calculate the loss. The binary cross-entropy loss is defined as follows:

(2) $$\begin{array}{c} L_{\text{BCE}}=-\left(y\times \text{log}\left(p\left(y\right)\right)+\left(1-y\right)\times \text{log}\left(1-p\left(y\right)\right)\right), \end{array}$$

where *y* is the binary label value and *p*(*y*) is the predicted probability of *y*. The Tversky loss function finds a better balance between recall and precision. The Tversky loss function is defined as follows:

(3) $$\begin{array}{c} L_{\text{Tversky}}\left(P,G;\alpha ,\beta \right)=\frac{\left|PG\right|}{\left|PG\right|+\alpha \left|p/G\right|+\beta \left|G/P\right|}, \end{array}$$

where *P* and *G* represent predicted and true values; the trade-off between false negatives (FN) and false positives (FP) can be controlled by adjusting the hyperparameters *α* and *β*. We set *α* to 0.3 and *β* to 0.7. The final model loss function is defined as follows:

(4) $$\begin{array}{c} \text{Loss}=\omega \times L_{\text{BCE}}+\left(1-\omega \right)\times L_{\text{Tversky}}, \end{array}$$

where *ω* is selected as 0.5 according to experience. The final constructed loss function can solve the class imbalance problem between foreground and background pixels.

## 4. Experimental Results

### 4.1. Evaluation Dataset and Metrics

We conducted experiments on the CDnet-2014 dataset [9] and evaluated the proposed method. The CDnet-2014 dataset contains 53 video sequences from real scenes, totaling nearly 160,000 frames, divided into 11 categories, corresponding to 11 different challenges: Camera Jitter, Baseline, Intermittent Object Motion, Dynamic Background, Bad Weather, Shadow, Night Videos, Low Framerate, PTZ, Thermal, and Turbulence. Each category has 4 to 6 video sequences, and the video resolution ranges from 320 × 240 to 720 × 576. The CDnet-2014 dataset covers a variety of challenging scenarios and is the most comprehensive dataset in the field of foreground detection.

The most commonly used quantitative evaluation indicators for foreground detection mainly include recall (Re), precision (Pr), false negative rate (FNR), false positive rate (FPR), specificity (Sp), F-measure (FM), and percentage of wrong classifications (PWC). Among them, F-measure represents the balance between recall and precision and is a comprehensive indicator that can best reflect the performance of different methods. Given true positive (TP), true negative (TN), false positive (FP), and false negative (FN), seven evaluation metrics are defined as follows:

(5) $$\begin{array}{c} \text{Recall }=\frac{\text{TP }}{\text{TP }+\text{ FN}},\\ \text{Precision }=\frac{\text{TP}}{\text{TP }+\text{ FP}},\\ \text{Specificity }=\frac{\text{TN}}{\text{ TN }+\text{ FP}},\\ \text{FNR }=\frac{\text{FN}}{\text{TP }+\text{ FN}},\\ \text{FPR }=\frac{\text{FP}}{\text{FP }+\text{ TN}},\\ \text{PWC }=\;\frac{100\;\times \left(\text{FN }+\text{ FP}\right)}{\text{TP }\;+\text{ FN }\;+\text{ FP }+\text{TN}},\\ F-\text{Measure}\;=\;\frac{2\times \text{Recall}\times \text{Precision}}{\text{Recall}\;+\;\text{Precision}}. \end{array}$$

### 4.2. Implementation and Training Details

Since the CDnet-2014 dataset is a test dataset, there is no training set or validation set, so we adopt the same strategy as FgSegNet_v2 [31] to manually select 200 frames from each video sequence as training data and the rest as test data. There are 53 video sequences in the dataset, and there are 10,600 training data images in total, accounting for about 6.65% of the total number of images in the dataset. All training and testing data are resized to a uniform size of 640 × 480, which is 640 in length and 480 in width. To demonstrate the effectiveness of our proposed model, our method does not use any preprocessing or post-processing procedures.

Table 1 shows the configurations of the proposed AMU-Net model, where “conv” means convolution operation, “maxpool” means max-pooling operation, “attention” means attention operation, and “tranconv” means transposed convolution operation.

The hardware platform of the experiment is based on an Intel Core i7-9700 8-core CPU and a single NVIDIA GeForce RTX 2080 Ti 11G GPU; the software environment is Windows 10 + Python 3.8 + PyTorch 1.10.0; the parallel computing framework is CUDA Toolkit 10.2; and the acceleration library is cuDNN 7.6.5. The weights of the VGG-16 module used in AMU-Net are initialized using pretrained weights on ImageNet. The network model parameters are updated using the Adam optimization algorithm during training, the momentum is set to 0.9 and 0.999, and the batch size is set to 4. The initial learning rate is set to 1e-4, a total of 40 epochs are trained, and the learning rate is reduced by a factor of 10 after every 20 epochs. 90% of the training data is used for training the model and 10% for validation. The upsampling layer is implemented by transposed convolution and is a trainable parameter. On the CDnet-2014 dataset, it takes about 12 hours (about 19 minutes per epoch) to complete the entire training process of AMU-Net.

### 4.3. Ablation Analysis

To prove the effectiveness of the attention mechanism in the design of this model, we conducted ablation experiments on AMU-Net, removed the attention mechanism module, and called this network model MU-Net, which is similar to the original U-Net, and the feature maps corresponding to the decoder and encoder are directly concatenated through skip connections. The same training strategy is used during training to ensure the credibility of the comparison results. We use precision, recall, and F-measure as comparison indicators. The specific experimental results are shown in Table 2. It can be seen from the experimental results that the performance of the model with the addition of the attention mechanism has been comprehensively improved. Because the attention mechanism can achieve the purpose of enhancing the target features and suppressing the background, it is helpful to improve the detection quality.

### 4.4. Comparison with State-of-the-Art Methods

#### 4.4.1. Quantitative Evaluation

To evaluate the performance of the proposed AMU-Net model, we conducted validation experiments on the CDnet-2014 dataset, and the results are shown in Table 3. In the Baseline, Camera Jitter, Dynamic Background, and Shadow categories, the F-measure score of AMU-Net is higher than 0.99, which shows that our model has a strong processing ability for camera jitter, dynamic background, and shadow scenes. In challenging categories like PTZ, Night Videos, Intermittent Object Motion, Bad Weather, Thermal, and Turbulence, the F-measure score is also around 0.98. Taken together, an F-measure score of 0.9785 and a PWC score of 0.0603 were obtained on the entire dataset. According to [28], if the F-measure score of an algorithm is higher than 0.94 and the PWC score is lower than 0.9, the foreground detection results of the algorithm can be considered as good as the benchmark results of manual labeling. Therefore, the segmentation accuracy of our model reaches manual labeling accuracy in most video scenes. The AMU-Net model does not perform as well in the Low Framerate category as other categories, because some video sequences (such as the port_0_17fps sequence) in the Low Framerate category contain extremely small foreground objects, and there are lighting changes and dynamic backgrounds in the scene. In this case, the features of foreground objects are difficult to extract effectively, and the model may pay more attention to the main category (background) and less attention to rare categories (foreground), resulting in the misclassification of very small foreground objects as background. The recall score is only 0.8921, so the F-measure score is low.

We compared the AMU-Net method with some classic and state-of-the-art methods, mainly comparing the F-measure metric scores on the CDnet-2014 dataset for different class sequences and overal dataset. We choose the following methods: GMM [14], FTSG [23], SuBSENSE [21], IUTIS-5 [25], DeepBS [27], CascadeCNN [28], BSPVGAN [39], and FgSegNet_v2 [31], where DeepBS, CascadeCNN, BSPVGAN, and FgSegNet_v2 are deep learning methods, and the rest are unsupervised traditional methods.  Table 4 presents a quantitative comparison of the F-measure scores, and the first, second, and third results are marked with bold red, blue, and green fonts, respectively. It can be seen that the top three F-measure scores are all based on deep learning, and the results are much higher than traditional methods, especially in very challenging categories (such as Night Videos and PTZ), which shows the advantages of deep learning in the foreground detection task. The performance of our proposed AMU-Net is close to the current state-of-the-art methods, significantly outperforming BSPVGAN and CascadeCNN, and only slightly worse than FgSegNet_v2. It should be noted that since FgSegNet_v2 was proposed, it has been ranked first on the CDnet-2014 official website, and its F-measure of most categories is close to 1, which is relatively difficult to improve. However, FgSegNet_v2 is a scene-specific method, which trains a network model separately for each scene video sequence, resulting in a total of 53 networks with different parameters. It requires more weight parameters and takes longer for training. On the other hand, our method only needs to train a general network model for 53 video sequences, the required weight parameters are reduced by nearly 20 times, and the training time is greatly shortened.  Table 5 shows the differences between AMU-Net and FgSegNet_v2.

#### 4.4.2. Qualitative Evaluation

**Figure 4:**
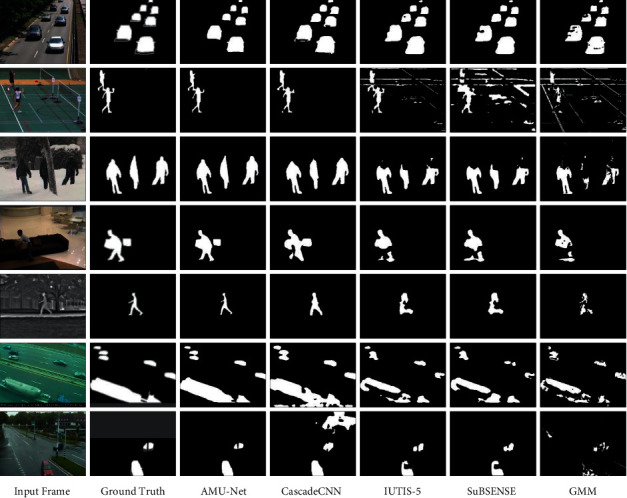
Qualitative comparison of the proposed AMU-Net and other models.

To evaluate the performance of our proposed method in different scenarios from a qualitative analysis point of view, we selected the following representative video sequences (without training frames) from the CDnet-2014 dataset for evaluation, covering several categories of typical challenges scenes: highway sequence in Baseline category (#820), badminton sequence in Camera Jitter category (#1139), skating sequence in Bad Weather category (#1910), sofa sequence in Intermittent Object Motion category (#2023), park sequence in Thermal category (#508), turnpike_0_5fps sequence in Low Framerate category (#1011), and twoPositionPTZCam sequence in PTZ category (#1013). The results are shown in Figure 4, where the first column shows the input frames, the second column shows the corresponding benchmark ground truth images, and the third to seventh columns show the following method results: our method (AMU-Net), CascadeCNN [28], IUTIS-5 [25], SuBSENSE [21], and GMM [14]. It can be seen visually that the results of our method significantly outperform all other methods, which is also consistent with the above quantitative evaluation results.

The first row is the highway sequence in the Baseline category. Our model can resist the interference of dynamic backgrounds (shaking branches) and shadows. The segmentation results are almost consistent with the ground truth. The foreground detection by the other methods is affected by shadows, often including shaded parts. The second line is the badminton sequence in the Camera Jitter category. The violent shaking of the camera causes the global motion of the video background. There is a lot of noise in the detection results of the traditional algorithm, but our model relatively completely segmented the foreground target. The third row is the skating sequence in the Bad Weather category. Our model detects the human head and torso very clearly in the scene but hardly sees the human head in the detection results of other methods. The fourth row is the sofa sequence in the Intermittent Object Motion category. The color and texture of the pants worn by the people in the picture are very similar to the sofa behind them, which leads to holes in the foreground detection results of other methods, while our model can detect the complete outline of the person well. In addition, it can be seen that the traditional algorithm absorbs the box on the sofa into the background, resulting in missed detection, while our method accurately detects the target. The fifth row is the park sequence in the Thermal category. Due to the serious loss of color information in the infrared image, in this case, it is difficult for even a person to accurately segment the foreground object from the background, but our method can also segment the pedestrian more accurately. The sixth row is the turnpike_0_5fps sequence in the Low Framerate category. The low frame rate leads to a large difference in foreground targets between adjacent frames. It can be seen that the segmentation results of the proposed model are the closest to the ground truth, while the results of other methods have a lot of noise and fail to detect foreground targets completely. The seventh row is the twoPositionPTZCam sequence in the PTZ category. The global motion caused by the nonstationary camera is not conducive to foreground segmentation. Only the proposed model can detect the moving car target perfectly.

Overall, the proposed method can perform well in the face of various interference challenges, because we introduce attention mechanisms in the multiscale structure, which improves the robustness of the model to various noises in the background so that the model can be more “focused” on the foreground targets of different sizes.

### 4.5. Multiple Input Experiments

**Figure 5:**
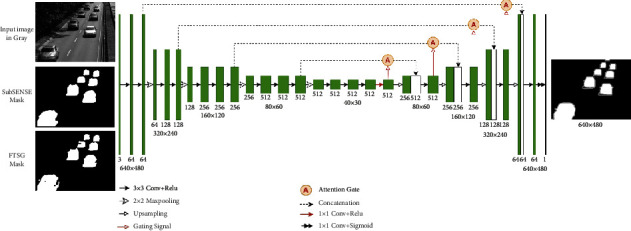
The architecture of the proposed AMU-Net_M1 for foreground detection.

**Figure 6:**
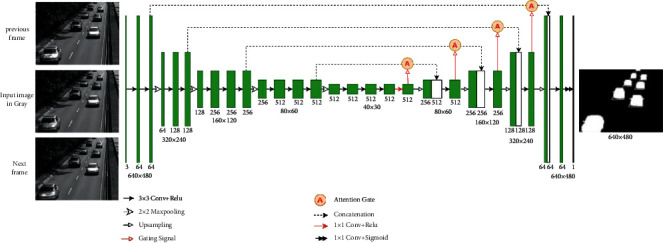
The architecture of the proposed AMU-Net_M2 for foreground detection.

Our proposed AMU-Net model only needs to input a single RGB image and only uses the spatial information of the image to segment the foreground target very well. In order to use the temporal information of the video to further improve the detection ability of the model, we considered two multiple input methods. The first is to refer to the method of [46], converting the input RGB image into a grayscale image as a spatial appearance cue, the segmentation mask of the SuBSENSE algorithm as a change cue, and the output of the FTSG algorithm as a motion cue, so that the original RGB three-channel input becomes a three-channel input of grayscale, change, and motion cues. In order to distinguish it from the single-input model, we call it AMU-Net_M1 (see Figure 5). The second is to convert the current input frame and its previous and next frames into grayscale images as three-channel input, called AMU-Net_M2 (see Figure 6). Through the fusion input of multiple images, more spatiotemporal information is included, and the deep learning framework can learn richer features from it, thereby achieving a more robust output.

We still conducted experiments and model evaluations on the CDnet-2014 dataset. We compare AMU-Net_M1, AMU-Net_M2, AMU-Net, SuBSENSE, and FTSG. The first is quantitative analysis, and the results are shown in Table 6. It can be seen that AMU-Net_M1 has the highest scores in the three categories of Intermittent Object Motion, Low Framerate, and Turbulence. Especially in the Low Framerate category, F-measure has increased from 0.9030 of AMU-Net to 0.9603, an increase of 6.35%, and even surpassed the score of FgSegNet_v2 in this category (0.9579), and the overall F-measure has increased to 0.9827. AMU-Net_M2 achieved the highest score in the four categories of PTZ, Baseline, Shadow, and Thermal, with a total score of 0.9830. The above results show that the input of multiple images allows the deep neural network to learn more abstract spatiotemporal features, thereby achieving more robust detection performance.

**Figure 7:**
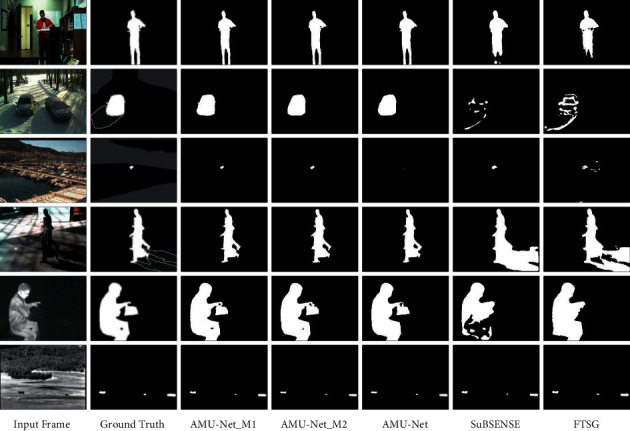
Qualitative comparison of different methods.

The qualitative evaluation results are shown in Figure 7. We selected the following video sequences (without training frames) from the CDnet-2014 dataset for evaluation: office sequence in the Baseline category (#1717), winterDriveway sequence in the Intermittent Object Motion category (#1826), port_0_17fps sequence in the Low Framerate category (# 1082), peopleInShade sequence in the Shadow category (#1085), library sequence in the Thermal category (#2615), and turbulence0 sequence in the Turbulence category (#2265). It can be seen that the AMU-Net_M1 and AMU-Net_M2 methods have the best detection results, which is consistent with the above quantitative analysis results. Especially in the port_0_17fps sequence in the Low Framerate category in the second row, the moving foreground target in the scene is extremely small, and it is also interfered with by the dynamic background and lighting. The AMU-Net model pays more attention to the background and ignores the foreground, so it is almost impossible to detect the foreground target. However, with the help of multiple inputs, the AMU-Net_M1 and AMU-Net_M2 models can better learn the relevant features of the foreground targets. So the detection results are basically the same as ground truth.

## 5. Conclusion

This paper proposed a foreground detection method based on the multiscale U-Net architecture with a fusion attention mechanism. Introducing the attention mechanism into the multiscale structure can increase the weight of foreground objects and suppress the interference of background pixels. The model only needs a small amount of data for training and only uses spatial information to achieve accurate foreground segmentation. We also conducted two types of multi-input experiments on the model. One is to input the foreground mask of the traditional unsupervised method SuBSENSE algorithm and FTSG algorithm together with the grayscale image of the input frame into the model, and the other is to input the current input frame and its previous and next frames which are converted into grayscale images as input. Through multiple inputs, the model can learn more motion and spatiotemporal features, which improves the robustness of model detection. We introduce the architecture of the model network in detail and set up a series of comparative experiments from both qualitative and quantitative perspectives using the CDnet-2014 dataset. The proposed foreground detection method outperforms many existing methods without any training tricks or post-processing, and the F-measure score is significantly improved compared to other methods. However, the generalization ability of the proposed method is not strong, and the real-time performance is slightly poor. For future work, we plan to further improve the model architecture, introduce more spatiotemporal information, and generalize the method to unseen videos.

## Figures and Tables

**Table 1:** The proposed AMU-Net model configurations.

Layer	Kernel	Stride	Channel	Output size
Input	—	—	3	640*∗*480
conv1_1	3*∗*3	1	64	640*∗*480
conv1_2	3*∗*3	1	64	640*∗*480
maxpool_1	2*∗*2	2	64	320*∗*240
conv2_1	3*∗*3	1	128	320*∗*240
conv2_2	3*∗*3	1	128	320*∗*240
maxpool_2	2*∗*2	2	128	160*∗*120
conv3_1	3*∗*3	1	256	160*∗*120
conv3_2	3*∗*3	1	256	160*∗*120
conv3_3	3*∗*3	1	256	160*∗*120
maxpool_3	2*∗*2	2	256	80*∗*60
conv4_1	3*∗*3	1	512	80*∗*60
conv4_2	3*∗*3	1	512	80*∗*60
conv4_3	3*∗*3	1	512	80*∗*60
maxpool_4	2*∗*2	2	512	40*∗*30
conv5_1	3*∗*3	1	512	40*∗*30
conv5_2	3*∗*3	1	512	40*∗*30
conv5_3	3*∗*3	1	512	40*∗*30
conv6	1*∗*1	1	512	40*∗*30
attention4	1*∗*1	1	512	80*∗*60
attention3	1*∗*1	1	256	160*∗*120
attention2	1*∗*1	1	128	320*∗*240
attention1	1*∗*1	1	64	640*∗*480
tranconv4	4*∗*4	2	256	80*∗*60
conv4d	3*∗*3	1	512	80*∗*60
tranconv3	4*∗*4	2	256	160*∗*120
conv3d	3*∗*3	1	256	160*∗*120
tranconv2	4*∗*4	2	128	320*∗*240
conv2d	3*∗*3	1	128	320*∗*240
tranconv1	4*∗*4	2	64	640*∗*480
conv1d	3*∗*3	1	64	640*∗*480
conv_out	1*∗*1	1	1	640*∗*480

**Table 2:** Results of ablation analysis.

Model	Precision	Recall	F-measure
MU-Net	0.9807	0.9684	0.9742
AMU-Net	**0.9850**	**0.9724**	**0.9785**

The bold values indicate the better result in a given column.

**Table 3:** Complete results of the AMU-Net on CDnet-2014 datasets.

Category	Precision	Recall	Specificity	FNR	FPR	PWC	F-measure
PTZ	0.9907	0.9628	0.9999	0.0372	0.0001	0.0285	0.9759
Bad Weather	0.9897	0.9853	0.9998	0.0147	0.0002	0.0425	0.9875
Baseline	0.9970	0.9903	0.9999	0.0097	0.0001	0.0328	0.9936
Camera Jitter	0.9937	0.9889	0.9997	0.0111	0.0003	0.0679	0.9913
Dynamic Bg	0.9965	0.9864	0.9999	0.0136	0.0001	0.0145	0.9914
Intermitt	0.9957	0.9805	0.9997	0.0195	0.0003	0.1613	0.9879
Low Framerate	0.9150	0.8921	0.9998	0.1079	0.0002	0.0475	0.9030
Night Videos	0.9860	0.9701	0.9997	0.0299	0.0003	0.0934	0.9779
Shadow	0.9922	0.9921	0.9996	0.0079	0.0004	0.0657	0.9921
Thermal	0.9907	0.9854	0.9995	0.0146	0.0005	0.0842	0.9880
Turbulence	0.9876	0.9630	0.9999	0.0370	0.0001	0.0256	0.9751
Overall	0.9850	0.9724	0.9998	0.0276	0.0002	0.0603	0.9785

**Table 4:** F-measure comparison of different methods on CDnet-2014 dataset.

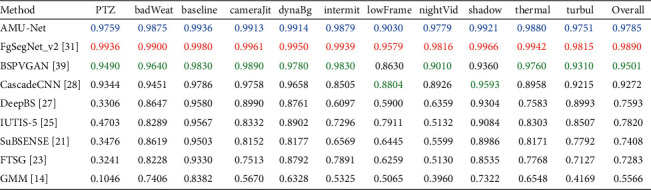

**Table 5:** The differences between AMU-Net and FgSegNet_v2.

Methods	Number of models	Network parameters	Training time	GPU
FgSegNet_v2 [31]	53	489 M (53*∗*9,225,161)	29 days [46]	1080 Ti
AMU-Net	1	24.9 M (24, 915, 969)	12 hours	2080 Ti

**Table 6:** F-measure comparison of different methods on CDnet-2014 dataset.

Method	PTZ	badWeat	Baseline	cameraJit	dynaBg	Intermit	lowFrame	nightVid	Shadow	Thermal	Turbul	Overall
AMU-Net_M1	0.9684	0.9865	0.9936	0.9884	0.9909	**0.9897**	**0.9603**	0.9733	0.9928	0.9898	**0.9764**	0.9827
AMU-Net_M2	**0.9759**	0.9829	**0.9941**	0.9873	0.9913	0.9888	0.9596	0.9741	**0.9930**	**0.9900**	0.9754	**0.9830**
AMU-Net	**0.9759**	**0.9875**	0.9936	**0.9913**	**0.9914**	0.9879	0.9030	**0.9779**	0.9921	0.9880	0.9751	0.9785
CascadeCNN [28]	0.9344	0.9451	0.9786	0.9758	0.9658	0.8505	0.8804	0.8926	0.9593	0.8958	0.9215	0.9272
SuBSENSE [21]	0.3476	0.8619	0.9503	0.8152	0.8177	0.6569	0.6445	0.5599	0.8986	0.8171	0.7792	0.7408
FTSG [23]	0.3241	0.8228	0.9330	0.7513	0.8792	0.7891	0.6259	0.5130	0.8535	0.7768	0.7127	0.7283
GMM [14]	0.1046	0.7406	0.8382	0.5670	0.6328	0.5325	0.5065	0.3960	0.7322	0.6548	0.4169	0.5566

## Data Availability

The data used to support the findings of this study are available from the corresponding author upon request.
